# Self‐Assembled Biomolecular 1D Nanostructures for Aqueous Sodium‐Ion Battery

**DOI:** 10.1002/advs.201700634

**Published:** 2018-01-03

**Authors:** Huiwu Long, Wen Zeng, Hua Wang, Mengmeng Qian, Yanhong Liang, Zhongchang Wang

**Affiliations:** ^1^ College of Materials Science and Engineering Chongqing University Chongqing 400044 China; ^2^ School of Chemistry and Environment Beihang University Beijing 100191 China; ^3^ Material Simulation and Computing Laboratory Department of Physics Hebei Normal University of Science and Technology Hebei 066004 China; ^4^ Department of Quantum Materials Science and Technology International Iberian Nanotechnology Laboratory (INL) Av. Mestre José Veiga s/n Braga 4715‐330 Portugal

**Keywords:** alizarin, aqueous electrolytes, biomolecules, self‐assembly, sodium‐ion batteries

## Abstract

Aqueous sodium‐ion battery of low cost, inherent safety, and environmental benignity holds substantial promise for new‐generation energy storage applications. However, the narrow potential window of water and the enlarged ionic radius because of hydration restrict the selection of electrode materials used in the aqueous electrolyte. Here, inspired by the efficient redox reaction of biomolecules during cellular energy metabolism, a proof of concept is proposed that the redox‐active biomolecule alizarin can act as a novel electrode material for the aqueous sodium‐ion battery. It is demonstrated that the specific capacity of the self‐assembled alizarin nanowires can reach as high as 233.1 mA h g^−1^, surpassing the majority of anodes ever utilized in the aqueous sodium‐ion batteries. Paired with biocompatible and biodegradable polypyrrole, this full battery system shows excellent sodium storage ability and flexibility, indicating its potential applications in wearable electronics and biointegrated devices. It is also shown that the electrochemical properties of electrodes can be tailored by manipulating naturally occurring 9,10‐anthroquinones with various substituent groups, which broadens application prospect of biomolecules in aqueous sodium‐ion batteries.

Sodium‐ion batteries are considered as an alternative to lithium‐ion batteries due to the natural abundance of sodium resource.^1^, ^2^, ^3^ Nevertheless, the high‐cost, flammable, and toxic organic electrolytes still hinder their applications as grid‐scale energy storage devices, electric vehicles, and portable electronics.^4^, ^5^ Replacing the organic electrolytes with inexpensive and environment friendly water,^6^, ^7^, ^8^ aqueous sodium‐ion batteries hence emerge as an attractive battery system.^9^, ^10^ However, differing from nonaqueous electrolyte, the potential window of water is narrower owing to the H_2_/O_2_ evolution, limiting the choice of electrode materials.^6^, ^9^ Meanwhile, the sodium ion in the aqueous electrolyte exists in a hydrated form with a larger radius, making the insertion/desertion process more difficult, which exerts detrimental impact on both kinetics and capacity.^11^ The exploration of novel electrode materials with a suitable redox potential and superb electrochemical properties is hence highly desirable yet extremely challenging.

In view of the intimate connection of life to water, reaction in aqueous environment is universal in life activity, which stimulates valuable inspiration to address the aforementioned issues.^12^ The shuttle of electrons during cellular metabolism relies on the redox reaction of biomolecules, and therefore the biomolecules with reversible redox activity are expected to possess potentials for applications as energy storage devices. More importantly, these redox reactions occur in tissue fluid, demonstrating that their redox potentials exactly fall in the potential window of water.^13^, ^14^, ^15^ In comparison to inorganic materials, the organic feature of biomolecules endows them a better structural flexibility, which is more favorable for the insertion/desertion of the enlarged hydrated sodium ion and thus may improve the kinetics and capacity.^11^, ^16^ Another characteristic of biomolecules rests with their chemical diversity resulting from the variety of substituent groups, which makes their electrochemical properties more adjustable.^13^ Importantly, widespread distribution of biomolecules in ecosystem makes them renewable and sustainable, adding further economic and environmental benefits to the battery systems.^12^, ^13^, ^14^, ^17^ In this sense, biomolecules might serve as a valuable material pool for electrode fabrication in aqueous sodium‐ion batteries.

As one of the typical biomolecules, Quinone derivatives have been proven to possess the electrochemical lithium ion storage ability.^18^, ^19^ Moreover, their redox potential is associated with the molecular backbone aromaticity. In comparison to the 1,4‐benzoquinone and 1,4‐naphthoquinone, 9,10‐anthraquinone has a redox potential that is closer to the H_2_ evolution threshold, suggesting its potential application as an anode in the aqueous electrolyte.^20^ Here, we propose a novel aqueous sodium‐ion battery system upon the 9,10‐anthroquinone alizarin (**Figure** **1**), a cheap and easily accessible raw material that can be extracted from the plant Rubia cordifolia L. This material has been widely used as edible pigment and herbal medicine since the Bronze Age. We demonstrate that the alizarin molecule shows a redox activity at a relatively low potential and can be further fabricated into nanowires via a self‐assembly process. The specific capacity of the alizarin nanowires reaches 233.1 mA h g^−1^ at a current density of 5 A g^−1^, which exceeds the majority of anodes reported so far. Paired with polypyrrole (PPy) which also possesses biocompatibility and biodegradability, we demonstrate that this full battery system when bended can still power a portable device, displaying its possible applications as wearable electronics and biointegrated devices. Except for alizarin, we also systematically probe a series of naturally occurring 9,10‐anthroquinones with tunable electrochemical properties and testify the broad suitability of the biomolecules in aqueous sodium‐ion batteries. Our work provides detailed investigation to the utilization of biomolecules in aqueous sodium‐ion batteries for the first time and offers new insights into the development of new‐generation energy storage system.

**Figure 1:**
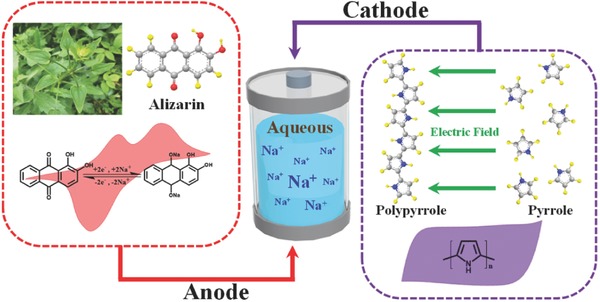
Sketch of aqueous sodium‐ion batteries with alizarin‐based anodes and polypyrrole‐based cathodes.

With aromatic feature, the alizarin molecule exhibits a considerable solubility in acetone yet is nearly insoluble in water. The antisolvent method was hence applied to synthesize the nanosized alizarin (**Figure** **2**a). Dissolved in acetone and dropped into water, change in solubility triggers recrystallization and self‐assembly of the alizarin molecule, during which the π–π interaction and hydrogen bonding are considered to play a critical role.^21^ The alizarin molecule possesses a planar configuration and the most stable assembly direction is vertical to the molecular plane, so it is inclined to form 1D nanostructure after the oriented growth.^22^, ^23^ Scanning electronic microscope (SEM) and transmission electronic microscope images confirm that these products are composed of 1D nanostructures and demonstrate that the diameter gradually decreases with the rise of the water/acetone volumetric ratio (Figure 2b–d; Figures S1 and S2, Supporting Information). When the ratio changes from 1 via 2.5 to 10, nanorods, nanoneedles, and nanowires are formed, respectively. Further Fourier transform infrared spectroscopy (FTIR) reveals that the antisolvent method does not change chemical composition of samples (Figure 2e). The peaks at 1666.5 and 1633.4 cm^−1^ refer to the stretching vibration of C=O at position 10 and 9, respectively, and the existence of two peaks of carbonyl groups is attributed to the intramolecular hydrogen bonding between the carbonyl group at the position 9 and the hydroxyl group at the position 1.^24^

**Figure 2:**
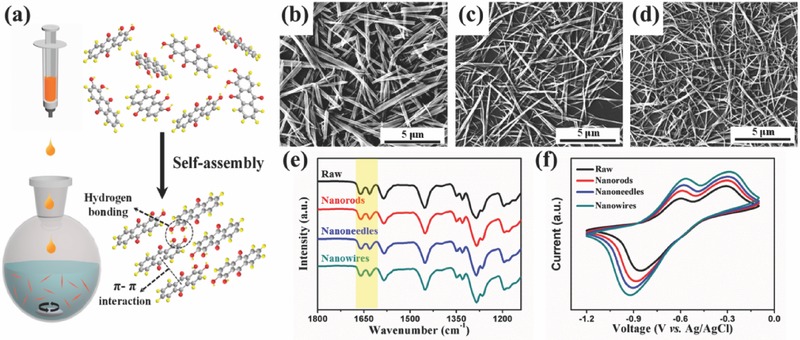
a) Schematical illustration of self‐assembly process of the alizarin. b–d) SEM images of 1D nanostructures with different diameters. e,f) FTIR spectra and CV curves of alizarin with different morphologies.

To shed light on electrochemical behavior of alizarin with different morphologies, cyclic voltammetry (CV) was tested in a three‐electrode system, where well‐defined redox peaks are observed in all samples, verifying the energy storage ability of alizarin (Figure 2f). Due to the fully conjugated cyclic diketone structure of the 9,10‐anthroquinone, the redox reaction can be generalized as enolization and its reverse process of the carbonyl group accompanied by the formation/decomposition of anthroquinone–Na complexes.^15^, ^25^ In comparison to the raw alizarin, the alizarin of 1D nanostructure displays higher peak currents and better sodium storage abilities, indicative of the superiority of 1D nanostructure in ion diffusion and electron transport.^21^ Furthermore, such a tendency turns more obvious with the decrease of diameter. Among all structures, the alizarin nanowires show the best electrochemical behavior, which will be selected for further study.

To gain insights into the electron transfer process and the charge storage mechanism of alizarin nanowires, we first present CV curves acquired using relatively low scan rate ranging from 1 to 20 mV s^−1^ (**Figure** **3**a). With the decrease of scan rate, two pairs of reversible redox peaks are observed obviously, indicating the emergence of two continuous single‐electron redox reactions for the alizarin nanowires. We also conducted density functional theory (DFT) calculations with a special focus on the highest occupied molecular orbital (HOMO)/lowest unoccupied molecular orbital (LUMO) energy levels of the alizarin molecule at different states (Figure 3b), where a lower LUMO energy level means a larger electron affinity, resulting in a higher reduction potential.^16^, ^21^, ^26^ The LUMO energy level of the alizarin molecule at the as‐prepared state is ≈4.3 eV lower than that at the semireduced state, implying occurrence of two distinct one‐electron redox reactions, in accordance with the CV results. The charge storage mechanism can also be clarified through the relationship between the peak current (*i*) and the scan rate (ν) in the CV curves, which can be simply expressed as *i* = *aν* ^b^.^27^, ^28^ If b = 1, the redox reaction is surface‐controlled, representing a capacitive process, while if b = 1/2, the redox reaction is diffusion‐controlled, involving typical battery materials like LiFePO_4_.^29^ We unveil a linear relation of the *i* to the square root of *v*, demonstrating that the reaction is diffusion‐controlled for alizarin in aqueous electrolyte (Figure 3c).

**Figure 3:**
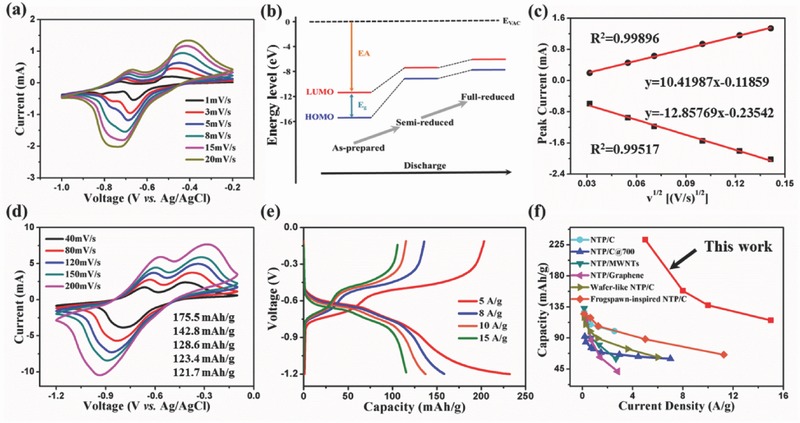
a) CV curves of alizarin nanowires obtained at relatively low scan rates. b) Energy level diagram of the alizarin molecule with different electronic states. c) The cathodic/anodic peak current as a function of the square root of scan rate. d) CV curves of alizarin nanowires at relatively high scan rates. e) Galvanostatic charging/discharging curves of alizarin nanowires at different current densities. f) Comparison of specific capacity of alizarin nanowires with those of widely used anodes including NaTi_2_(PO_4_)_3_ (NTP)/C, NTP/C@700, NTP/multiwalled carbon nanotubes (MWNTs), NTP/graphene, wafer‐like NTP/C, and frogspawn‐inspired NTP/C.

To estimate the rate capability of alizarin nanowires, we further measure the CV curves using relatively high scan rates varying from 40 to 200 mV s^−1^ (Figure 3d). With the rise of scan rate, the reduction peak moves to lower voltage while the oxidation peak shows the opposite, which is attributed to the insulation of the biomolecule and can be explained by the large energy gap (*E*_g_) of alizarin (Figure 3b).^30^ Albeit the polarization, the linear relationship retains between *i* and square root of *v* (Figure S3, Supporting Information), implying the superb sodium storage ability of alizarin nanowires at the high scan rate. Specific capacities are 175.5, 142.8, 128.6, 123.4, and 121.7 mA h g^−1^ at scan rates of 40, 80, 120, 150, and 200 mV s^−1^, respectively, indicative of excellent rate capacity (Figure 3d). Similar conclusions are drawn through the galvanostatic charging/discharging test, where the specific capacity is 233.1 mA h g^−1^ at the current density of 5 A g^−1^ and maintains at 115.8 mA h g^−1^ even at the high current density of 15 A g^−1^ (Figure 3e). Due to the narrow potential window of water and the enlarged ionic radius caused by hydration, suitable electrode materials for aqueous sodium‐ion batteries are limited and the NaTi_2_(PO_4_)_3_ (NTP)‐based materials are often chosen as anodes. However, our alizarin nanowires display a high specific capacity, which exceeds most NTP‐based anodes reported so far (Figure 3f),^31^, ^32^, ^33^, ^34^, ^35^, ^36^ therby encouraging us to further probe their electrochemical behavior in a full battery system.

To match alizarin‐based anode, a cathode that simultaneously possesses electrochemical and biological property is preferred. In addition to biocompatibility^37^ and biodegradability,^38^ PPy also shows superior electrochemical properties in a positive voltage range and can hence be selected as cathode (Figure S4, Supporting Information). The well‐defined redox peaks demonstrate an excellent sodium storage ability of this full battery system with alizarin nanowires as anode and PPy as cathode (**Figure** **4**a). The initial specific capacity on the basis of the active mass of alizarin nanowires is 146.1 mA h g^−1^ and maintains to be 65.1 mA h g^−1^ when the scan rate rises from 20 to 200 mV s^−1^ (Figure 4b). Subsequent galvanostatic charging/discharging test shows a sloping plateau with an average discharge potential of ≈1.06 V. In line with the CV curves, the discharge capacity is 151.8 mA h g^−1^ at the current density of 1 A g^−1^, and is still 62.8 mA h g^−1^ at the current density up to 5 A g^−1^ (Figure 4c). The full battery system has a considerably high specific capacity, albeit that the capacity retention needs to be improved (Figure S5, Supporting Information). Since decay curve of the full battery system almost has the same fitted equation to that of the anode alone, the poor cycling stability of the anode may directly be responsible for the undesirable capacity retention of the whole battery system, which may further be ascribed to the dissolution of reduced anthraquinone–Na complexes in water.^15^ It is worthy of noting that the cycling stability of electrode materials in aqueous electrolyte is a common issue that has not been completely solved yet.^9^

**Figure 4:**
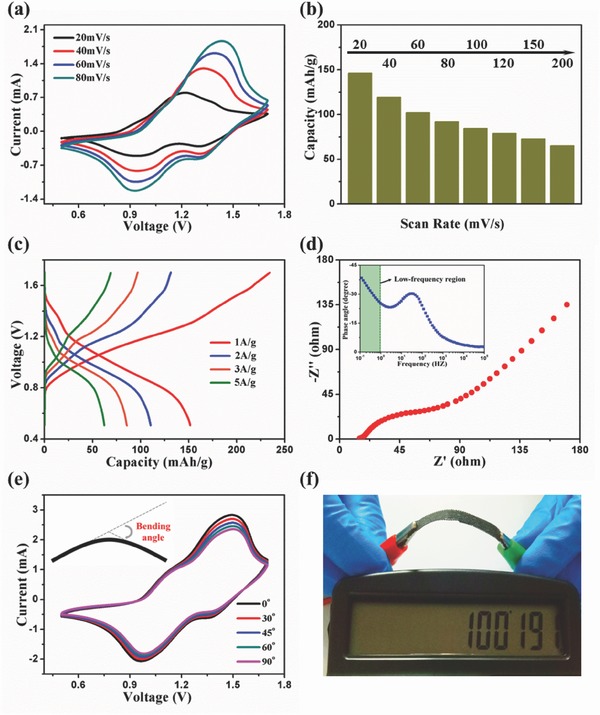
a) CV curves of full batteries at different scan rates. b) Specific capacity of full batteries against the san rate. c) Galvanostatic charging/discharging curves of full batteries at different current densities. d) Nyquist plot of full batteries. The inset shows the plot of the impedance phase angle versus frequency for the full batteries. e) CV curves of full batteries under different bending states at a scan rate of 100 mV s^−1^. f) Photograph showing the full batteries powering a calculator.

The electrochemical impedance spectroscope of the full battery system is composed of a semicircle in the high‐frequency region and a sloping line in the low‐frequency region (Figure 4d).^39^, ^40^, ^41^ The former portion is associated with the charge‐transfer impedance across the electrode/electrolyte interface and the latter at ≈45° to real axis is called Warburg tail involving the sodium‐ion diffusion in the electrode. The relatively low impedance of the sodium‐ion diffusion in the electrolyte, interface, and electrode is ascribed to high conductivity of the aqueous electrolyte and structural feasibility of alizarin. Flexibility is another crucial factor for the battery system due to the increasingly harsh service condition especially when it is developed as portable devices.^42^, ^43^ To test the flexibility of this full battery system, CV is first measured at different bending angles under a scan rate of 100 mV s^−1^. Nearly identical curves are observed, which shows an outstanding flexibility of the battery system (Figure 4e). Meanwhile, this full battery system can successfully power a calculator at bending condition (Figure 4f), indicative of its promising application in wearable electronics.

Due to the structural diversity of the biomolecule and the discovery of a variety of 9,10‐anthroquinones in plants (**Figure** **5**), alizarin is not the only candidate for aqueous sodium‐ion batteries. With different substituent groups at the position α (1, 4, 5, and 8) and β (2, 3, 6, and 7), these 9,10‐anthroquinones exhibit adjustable electrochemical properties.^15^, ^39^, ^40^ As an electron‐donating group, the hydroxyl group exists in almost all naturally occurring 9,10‐anthroquinones except the tectoquinone (6). When the hydroxyl group is introduced to the position β, electrons are injected into the π‐conjugated system, which increases LUMO energy level, resulting in a negative shift of the reduction potential. Conversely, when the hydroxyl group is introduced to the position α, there occurs an unusual positive shift of the reduction potential. This is attributed to the formation of the intramolecular hydrogen bonding between hydroxyl groups at the position α and carbonyl groups at the position 9 or 10, which could be detected by the FTIR spectra (Figure 2e).

**Figure 5:**
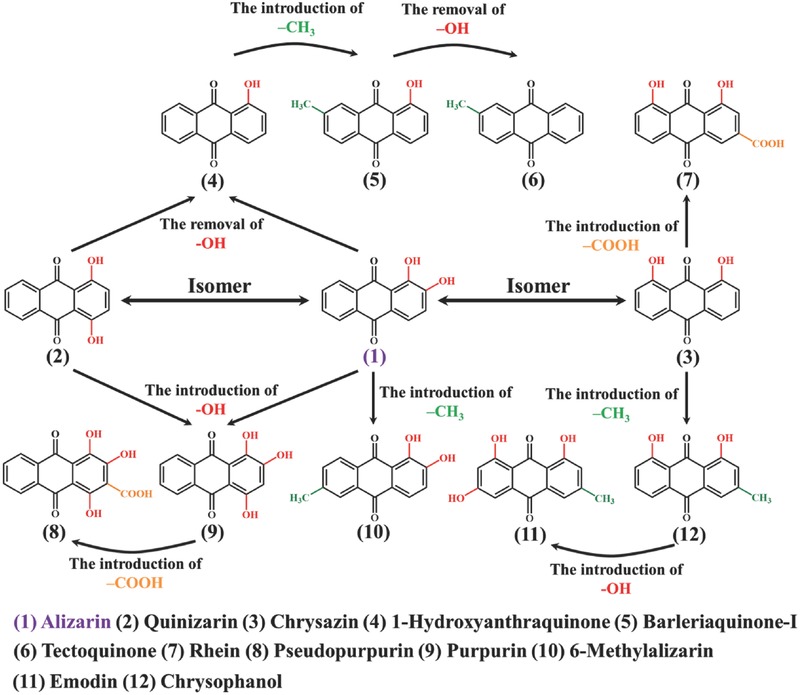
Family of naturally occurring 9,10‐anthroquinones with different substituent groups.

On the one hand, theoretically, quinizarin (2) shows a higher reduction potential than alizarin (1) because the number of intramolecular hydrogen bonding indisputably determines the extent of positive shift.^44^ On the other hand, how the intramolecular hydrogen bonding spatially distributes also plays a vital role. In view of electron delocalization, hydroxyl groups dispersed at different rings are stable, thus promoting the positive shift.^45^ Accordingly, the chrysazin (3) has a higher theoretical reduction potential than the quinizarin (2). In addition to the hydroxyl group, there usually exist carboxyl and methyl groups at the position β. For the carboxyl group, electrons are extracted from the π‐conjugated system, leading to positive shift of the reduction potential. By contrast, the methyl group represents another electron‐donating group that can cause negative shift of reduction potential. Another superiority of the methyl group is that it could decrease the solubility of biomolecules in the aqueous electrolyte, which is beneficial to the improvement of cycling stability. The combination of these substituent groups therefore helps to increase variability of naturally occurring 9,10‐anthroquinones with tunable reduction potential, solubility, and specific capacity (depending on molecular weight), manifesting their extensive application prospect in the aqueous sodium‐ion battery.

Summing up, we have successfully fabricated a new aqueous sodium‐ion battery system based on the biomolecule alizarin, a representative 9,10‐anthroquinone that can be extracted from plants. The alizarin molecule displays reversible redox peaks at a relatively low potential and can be synthesized into 1D nanostructure via the π–π interaction and hydrogen bonding. We find that the self‐assembled alizarin nanowires show a superb sodium storage ability, which exceeds most of the anodes used in aqueous sodium‐ion batteries so far. By applying the alizarin nanowires as anode and PPy as cathode, we fabricate a full battery system with superior specific capacity and flexibility, which can potentially be applied in the wearable electronics and biointegrated devices. We also demonstrate that the reduction potential, solubility and specific capacity can simply be adjusted by manipulating naturally occurring 9,10‐anthroquinones with diverse substituent groups, further enriching selectivity of electrode materials used in aqueous electrolyte. These findings open up a novel avenue in utilizing biomolecules in aqueous sodium‐ion batteries, which would facilitate the development of new‐generation energy storage system with a broad range of applications.

## Experimental Section

*Sample Preparation and Characterization*: In a typical synthesis process, 60 mg alizarin (Acros, purity 97%) was dissolved in 180 mL acetone, which was subsequently dropped into deionized water of different volumes under magnet stirring condition. The precipitation was eventually collected by centrifugation and dried at 50 °C overnight. Morphology of alizarin was observed by a field‐emission SEM (JSM‐7600F, JEOL) at a working voltage of 5 kV, and the Fourier transform infrared spectroscopy spectra were measured on a Nicolet iN10 spectrophotometer using transmission mode. DFT calculations were conducted using the Gaussian 09 software. A total of five geometries were optimized for each molecule by employing the restricted B3LYP hybrid functional and 6–311+G(d,p) basis set. The molecular orbital of each molecule, e.g., HOMO and LUMO energies, was analyzed.

*Property Measurement*: All electrochemical measurements were tested using a CHI 660D electrochemical workstation. The alizarin anode was prepared by mixing the active material, conductive material (acetylene black), and binder (polyvinyl alcohol, PVA) in a weight ratio of 5:3:2 using deionized water as dispersant agent, followed by a coating on carbon cloth. The PPy cathode was prepared through the electropolymerization of pyrrole (3.5 mg L^−1^) on the carbon cloth using p‐toluene sulfonic acid (10 mg L^−1^) as the dopant agent. The gel electrolyte was prepared by mixing 2.2 g PVA powder and 1.4 g NaClO_4_ in 10 mL H_2_O, followed by stirring at 80 °C until the solution turned transparent. The electrochemical characterization of anode or cathode alone was carried out in a standard three‐electrode system with a Pt wire as the counter electrode and a saturated Ag/AgCl electrode as the reference electrode. To fabricate the full battery, the alizarin anode was first placed on the glass substrate. Then, a piece of filter paper which had been soaked in the NaClO_4_ gel electrolyte for 30 min was put on the anode, serving as a separator to avoid the short circuit. Finally, the PPy cathode was placed on the top. Subsequently, the full battery was peeled of the glass substrate to form a flexible device. The electrochemical characterization of full batteries was conducted in a two‐electrode system.

## Conflict of Interest

The authors declare no conflict of interest.

## Supporting information

Supplementary
