# Supplementary material for: Self‐Assembled Biomolecular 1D Nanostructures for Aqueous Sodium‐Ion Battery
Source: Adv Sci (Weinh). 2018 Jan 3;5(3):1700634. doi: 10.1002/advs.201700634 (PMC5867053; doi:10.1002/advs.201700634)
Supplement: Supplementary file 1 — Supplementary [file ADVS-5-1700634-s001.pdf]

Copyright WILEY-VCH Verlag GmbH & Co. KGaA, 69469 Weinheim, Germany, 2018.

ADVANCED SCIENCE
Open Access

# Supporting Information

for *Adv. Sci.,* DOI: 10.1002/advs.201700634

# Self-Assembled Biomolecular 1D Nanostructures for Aqueous Sodium-Ion Battery

*Huiwu Long, Wen Zeng,* Hua Wang,* Mengmeng Qian, Yanhong Liang, and Zhongchang Wang**

Copyright WILEY-VCH Verlag GmbH & Co. KGaA, 69469 Weinheim, Germany, 2016.

# Supporting Information

## Self-Assembled Biomolecular 1D Nanostructures for Aqueous Sodium-Ion Battery

*Huiwu Long, Wen Zeng,* Hua Wang,* Mengmeng Qian, Yanhong Liang, and Zhongchang Wang**

### Calculation methods:

When tested by the cyclic voltammetry (CV) experiments, the capacity of electrodes (full battery system) was calculated through the integral area of the CV curve:

$$Q = \frac{1}{3.6v}\int_{V-}^{V+} i(V) \times dV \ ,$$

where $v$ is the scan rate.

On the other hand, when tested by the galvanostatic charging/discharging experiments, the capacity of electrodes (full battery system) was calculated by the following expression:

$$Q = \frac{It}{1.8} \ ,$$

where $I$ is discharging current and $t$ is discharging time. The specific capacity is obtained by dividing the capacity to the mass.

**Figure S1**

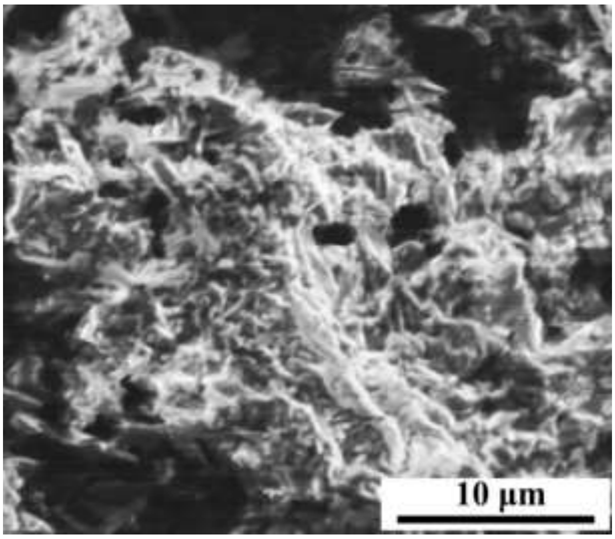


**Figure S1.** SEM image of the raw alizarin.

**Figure S2**

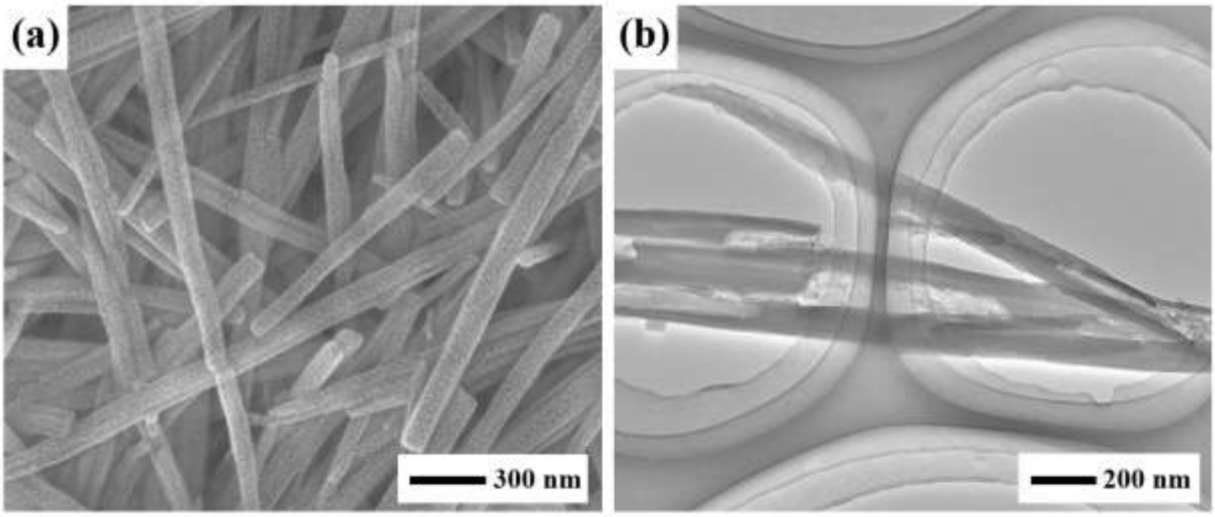


**Figure S2.** (a) SEM image and (b) TEM image of alizarin nanowires. The 1D nanostructure of alizarin could be further testified through the field emission scanning electron microscopy (FESEM) and transmission electron microscopy (TEM). Before the SEM measurement, gold spray was carried out to ensure electrical conductivity.

**Figure S3**

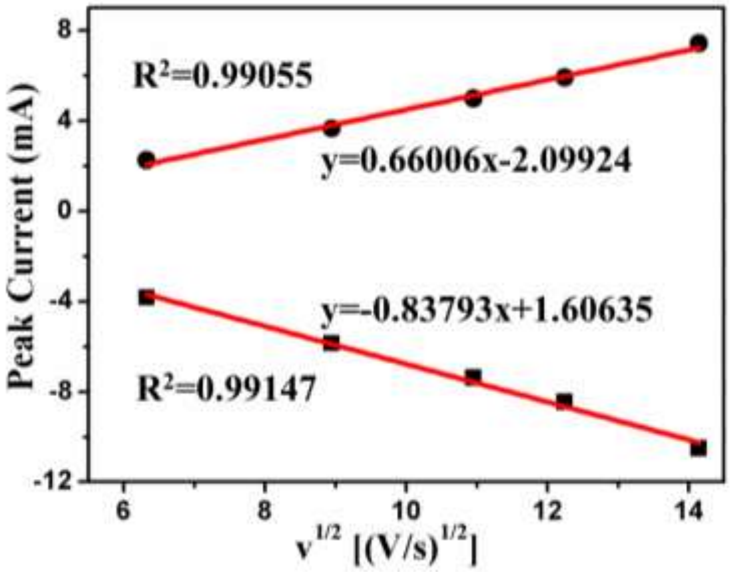


**Figure S3.** The relationship between the cathodic/anodic peak current and the square root of the scan rate (from 40 to 200 mV/s).

**Figure S4**

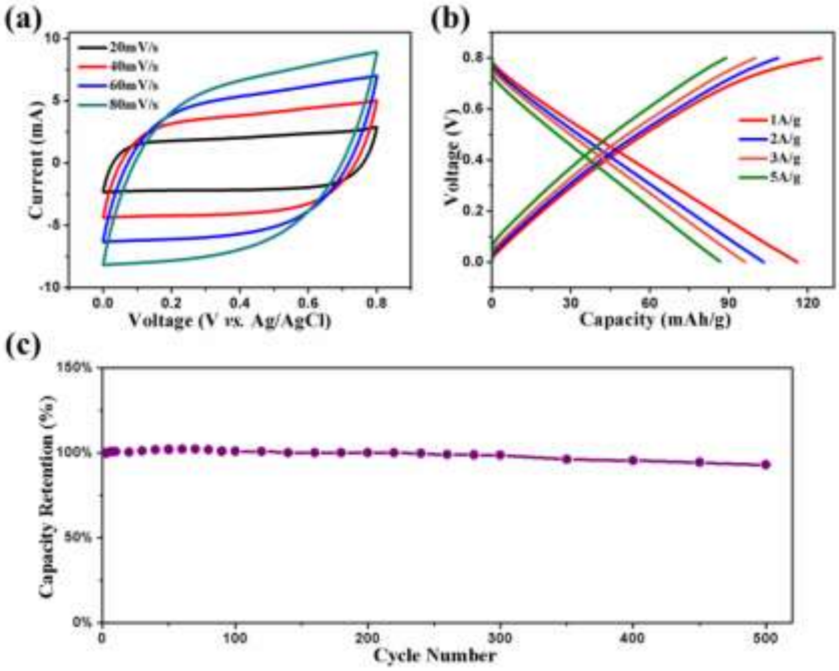


**Figure S4. (a)** CV curve of PPy at different scan rate. **(b)** Galvanostatic charging/discharging curves of PPy at different current density. **(c)** The cycle performance of PPy measured by CV at a scan rate of 100 mV/s. The PPy shows the superior sodium storage ability in this aqueous electrolyte. When measured by the galvanostatic charging/discharging test in a three-electrode system, the specific capacity is measured to be 116.1, 103.2, 96.1 and 86.7 mAh/g at a current density of 1, 2, 3 and 5 A/g, respectively. The specific capacity almost keeps unchanged after 500 cycles.

**Figure S5**

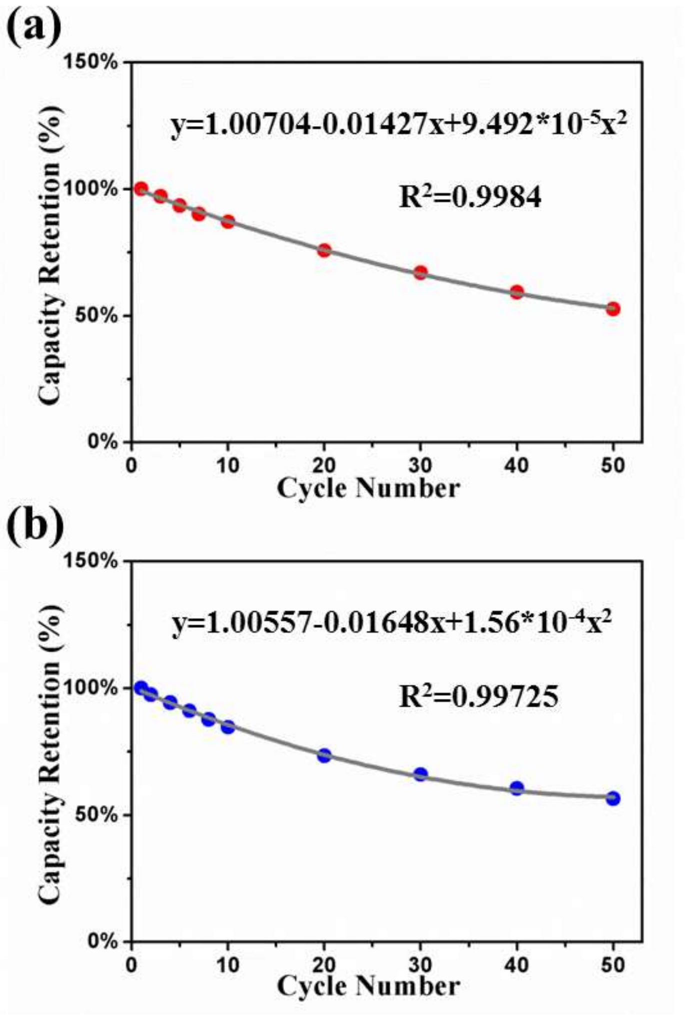


**Figure S5.** Cycle performance of **(a)** full batteries and **(b)** alizarin nanowires measured by CV at a scan rate of 100 mV/s. The inset shows the fitted equation of the decay curve.
